# Body Composition to Define Prognosis of Cancers Treated by Anti-Angiogenic Drugs

**DOI:** 10.3390/diagnostics13020205

**Published:** 2023-01-05

**Authors:** Pierre Decazes, Samy Ammari, Antoine De Prévia, Léo Mottay, Littisha Lawrance, Younes Belkouchi, Baya Benatsou, Laurence Albiges, Corinne Balleyguier, Pierre Vera, Nathalie Lassau

**Affiliations:** 1 Department of Medical Imaging and Nuclear Medicine, Henri Becquerel Cancer Center, 76038 Rouen, France; 2 QuantIF-LITIS (EA [Equipe d’ Accueil] 4108), Faculty of Medicine, University of Rouen, 76000 Rouen, France; 3 Biomaps, UMR1281 INSERM, CEA, CNRS, University of Paris-Saclay, 94805 Villejuif, France; 4 Department of Radiology, Gustave Roussy Cancer Campus, Université Paris-Saclay, 94800 Villejuif, France; 5 CVN, CentraleSupélec, Inria, Université Paris-Saclay, 91190 Gif-Sur-Yvette, France; 6 Department of Cancer Medicine, Gustave Roussy Cancer Campus, Université Paris-Saclay, 94800 Villejuif, France

**Keywords:** body composition, deep learning, angiogenesis inhibitor, computed tomography, muscle, adipose tissue, molecular targeted therapy

## Abstract

Background: Body composition could help to better define the prognosis of cancers treated with anti-angiogenics. The aim of this study is to evaluate the prognostic value of 3D and 2D anthropometric parameters in patients given anti-angiogenic treatments. Methods: 526 patients with different types of cancers were retrospectively included. The software Anthropometer3DNet was used to measure automatically fat body mass (FBM3D), muscle body mass (MBM3D), visceral fat mass (VFM3D) and subcutaneous fat mass (SFM3D) in 3D computed tomography. For comparison, equivalent two-dimensional measurements at the L3 level were also measured. The area under the curve (AUC) of the receiver operator characteristics (ROC) was used to determine the parameters’ predictive power and optimal cut-offs. A univariate analysis was performed using Kaplan–Meier on the overall survival (OS). Results: In ROC analysis, all 3D parameters appeared statistically significant: VFM3D (AUC = 0.554, *p* = 0.02, cutoff = 0.72 kg/m^2^), SFM3D (AUC = 0.544, *p* = 0.047, cutoff = 3.05 kg/m^2^), FBM3D (AUC = 0.550, *p* = 0.03, cutoff = 4.32 kg/m^2^) and MBM3D (AUC = 0.565, *p* = 0.007, cutoff = 5.47 kg/m^2^), but only one 2D parameter (visceral fat area VFA2D AUC = 0.548, *p* = 0.034). In log-rank tests, low VFM3D (*p* = 0.014), low SFM3D (*p* < 0.0001), low FBM3D (*p* = 0.00019) and low VFA2D (*p* = 0.0063) were found as a significant risk factor. Conclusion: automatic and 3D body composition on pre-therapeutic CT is feasible and can improve prognostication in patients treated with anti-angiogenic drugs. Moreover, the 3D measurements appear to be more effective than their 2D counterparts.

## 1. Introduction

Targeted therapy is a type of cancer treatment that targets proteins that control how cancer grows, divides, and spreads. This type of treatment has significantly improved outcomes across a wide range of solid tumors and, among them, antiangiogenic treatments, which block angiogenesis, are widely used [1,2].

Until now, there have been no global validated predictive factors that could accurately determine whether a patient would benefit from treatment with targeted antitumor agents, even if many groups have identified prognostic factors for some cancers and treatments. For metastatic kidney cancer, for example, clinical criteria (such as KPS <80% or a disease-free interval below 1 year) or biological criteria (such as elevated blood calcium, elevated LDH or anemia) can be combined to determine risk groups [3,4,5,6].

Among the recent biomarkers that aim to determine the prognosis of cancer patients, body composition parameters are promising. A global parameter for body composition is the body mass index (BMI) calculated from height and weight according to the following formula: BMI = weight/height^2^ (in kg/m^2^). It allows for evaluating the weight status, where a BMI ≥ 25 is considered overweight. Therefore, in a large population of metastatic melanoma treated with targeted therapy, immunotherapy, or chemotherapy, obesity was associated with improved survival and this association was mainly seen in male patients treated with targeted or immune therapies [7]. BMI is also a prognostic factor in metastatic colorectal cancers treated by targeted or non-targeted therapy, with better survival in case of higher BMI [8]. Comparable results were observed for renal cell carcinoma (RCC) patients treated with cabozantinib: A BMI ≥ 25 kg/m^2^ was correlated with longer survival [9]. Moreover, in metastatic RCC after one prior VEGFR-TKI therapy, everolimus is an effective treatment with the greatest benefit seen in patients with an age ≥ 65 years or with BMI >25 kg/m^2^ [10].

Although BMI is an interesting parameter to describe the weight status of patients, it does not describe body composition, such as fat and muscle compartments. Until now, these different compartments have often been estimated by a 2D method using a CT scan at the L3 abdominal level. These compartments can provide more information than the simple BMI: for instance, low BMI and sarcopenia are associated with dose-limiting toxicity of sorafenib in patients with renal cell carcinoma [11]. However, the 2D measurements seemed less accurate than their 3D multi-slice counterparts [12]. For example, it has been shown that during weight loss, changes in visceral and subcutaneous adipose tissue are poorly evaluated on 2D imaging [13], while 3D imaging gives good results for intra-abdominal fat [14]. Therefore, multi-slice segmentation is preferable [15], but needs automatic processing to avoid a time-consuming manual segmentation [13].

To analyze body composition, we developed a software, called Anthropometer3DNet, which allows the automatic and multi-slice measurement of anthropometric parameters on computed tomography (CT), routinely used for cancer patients. Initially developed to work on CT of PET/CT with a multi-atlas segmentation method [16], it has been improved by using neural networks and is now able to analyze diagnostic CT with variable acquisition fields (abdominal–pelvic or thoracic–abdominal–pelvic). This software can measure fat body mass (FBM_3D_), muscle body mass (MBM_3D_), visceral fat mass (VFM_3D_) and subcutaneous fat mass (SFM_3D_) automatically as whole-body parameters.

The main objective of this study is to evaluate the prognostic value of the 3D anthropometric parameters, evaluated automatically on CT scans using Anthropometer3DNET, and their equivalent 2D body composition parameters.

## 2. Materials and Methods

### 2.1. Population

Patients treated with anti-angiogenic treatments from 2003 to 2017 were included in this retrospective cohort [17]. These patients were treated for metastatic breast cancer, metastatic melanoma, metastatic colon cancer, metastatic gastro-intestinal stromal tumor (GIST), metastatic renal cell carcinoma (RCC), primary hepatocellular carcinoma (HCC) or other cancer. They were enrolled in a clinical trial of antiangiogenic-based therapy or were otherwise eligible to therapy with an approved antiangiogenic treatment. All patients provided written informed consent, either specific to this study or in the context of a clinical trial. The study was approved by the ethics committee of our institution and was declared to the French Commission Nationale Informatique et Liberté (CNIL MR-004).

### 2.2. Endpoints and Assessments

The following baseline clinical data were collected: age, sex, type of cancer, line of treatment, type of treatment. A diagnostic abdomen–pelvis or thorax–abdomen–pelvis CT scan was taken for all patients before starting targeted therapy. The CT scans were non-injected or acquired during the portal phase of the injection. The primary endpoint was overall survival (OS), defined as the time from the beginning of targeted therapy to death or last follow-up.

### 2.3. Anthropometric Parameters

The parameters were extracted by Anthropometer3DNet, an updated version of Anthropometer3D [16,18,19]. This software, usable for research purposes on the site www.oncometer3d.com, automatically measures in less than 5 min parameters FBM_3D_, MBM_3D_, VFM_3D_ and SFM_3D_ (in kg) on the CT of injected or non-injected (thoraco–)abdomino–pelvic CT. 

This software performs a deep learning-based segmentation of fat (visceral and subcutaneous) and muscle voxels based on a multi-slice 2D U-net Algorithm [20,21]. In parallel, it determines the slice levels using a Densenet Algorithm [21]. For parts outside the acquisition area, it uses adaptive extrapolation factors [22] for the tissues of interest (*k_muscle_* for muscles, *k_subcutaneous fat_* for subcutaneous fat and *k_visceral fat_* for visceral fat). These adaptive extrapolation factors are calculated on CT atlases as the mean ratio of whole-body voxels of muscle (or subcutaneous or visceral fat) divided by the numbers of voxels of muscle (or subcutaneous or visceral fat) in the acquired body area.

From the three types of voxels (visceral fat, subcutaneous fat, muscle), MBM_3D_, FBM_3D_, VFM_3D_ and SFM_3D_ are calculated as follows:

(1) $$MBM_{3D}{=N}_{muscle}\times k_{muscle}\times V_{voxel}\times \rho_{muscle}$$

(2) $$SCFM_{3D}{=N}_{subcutaneousfat}\times k_{subcutaneousfat}\times V_{voxel}\times \rho_{fat}$$

(3) $$VFM_{3D}{=N}_{visceralfat}\times k_{visceralfat}\times V_{voxel}\times \rho_{fat}$$

(4) $$FBM_{3D}=VFM_{3D}+SCFM_{3D}$$

With *N_muscle_*, *N_visceral_*_fat_ and *N_subcutaneous fat_* being the number of voxels of muscle, visceral fat and subcutaneous fat, respectively, obtained on the CT, *V_voxel_* the volume of one voxel (in ml). The density of the muscle (*ρ_muscle_*) was equal to 1.06 g/mL [23] and density of fat (*ρ_fat_*) was equal to 0.923 g/mL [24]. All values measured by the software were divided by the square of the patient’s body height (m^2^).

For comparison, the cross-sectional area at the level of the third lumbar vertebra of muscle body area (MBA_2D_), visceral fat area (VFA_2D_), subcutaneous fat area (SFA_2D_) and fat body area (FBA_2D_), combining SFA_2D_ and VFA_2D_, were automatically segmented by a 2D deep learning algorithm and normalized for stature (cm^2^/m^2^).

### 2.4. Statistical Analysis 

Descriptive statistics of the population and results were performed with continuous variables reported as mean ± standard deviation (SD) and categorical variables as frequencies (percentage). Correlations between each anthropometric parameter were evaluated using Spearman’s correlation coefficient. The predictive accuracy of survival at 1 year by anthropometric parameters was assessed by the receiver operator characteristics (ROC) analysis and measured by the area under the curve (AUC). An optimal cut-off value was computed by simultaneously maximizing specificity and sensitivity criteria (using Youden’s index). Two-sided tests were reported at the 5% level of significance. For parameters with an AUC statistically superior to 0.5, the Kaplan–Meier method was used to estimate the survival functions, and log-rank test was computed to evaluate the significance. Cox univariate proportional hazards models were used to test the relationship between study variables and survival rates for the global population, for men and for women. Finally, a subgroup analysis according to cancer type was performed.

## 3. Results

### 3.1. Population

A total of 526 patients were included in this retrospective study, their characteristics, including tumor types and anti-angiogenic treatments received, are described in Table 1.

### 3.2. Survival Analysis

**Figure 1:**
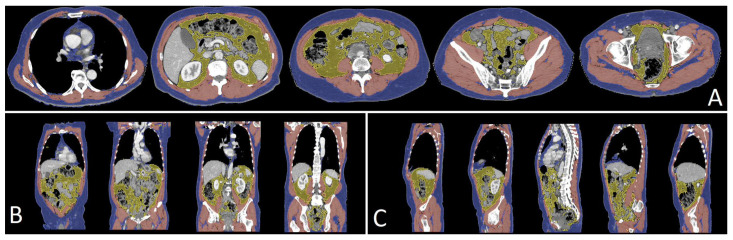
Results of the segmentation performed by Anthropometer3DNet of a patient in axial (**A**), frontal (**B**) and sagittal (**C**) views with, in blue, the subcutaneous tissue, in yellow the visceral adipose tissue and in red the muscle. For this patient, MBM_3D_ = 20.3 kg, FBM_3D_ = 15.3 kg, SAT_3D_ = 11.9 kg and VAT_3D_ = 3.4 kg.

A graphical representation of the automatic segmentation, of a patient’s pretreatment CT scan, is displayed in Figure 1.

**Figure 2:**
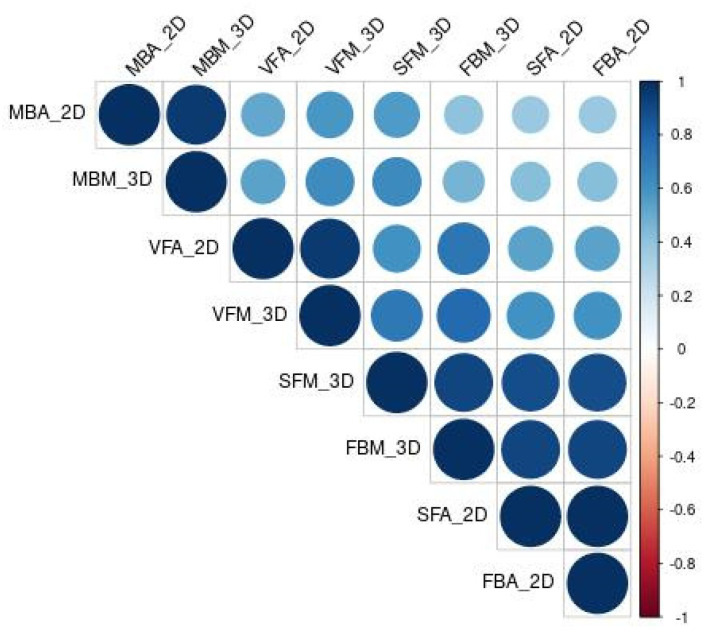
Correlation heatmap of Spearman’s correlation coefficients between the anthropometric parameters (VFA_2D_, SFA_2D_, FBA_2D_, MBA_2D_, VFM_3D_, SFM_3D_, FBM_3D_, MBM_3D_). The bigger the circle, the stronger the correlation.

The correlation coefficients between the different anthropometric parameters are presented in Figure 2. Multiple 2D measurements were correlated with their 3D counterparts: the coefficients of the parameters related to the muscle (MBA_2D_, MBM_3D_), visceral fat (VFA_2D_, VFM_3D_), and to subcutaneous/total fat (FBA_2D_, FBM_3D_, SFA_2D_, SFM_3D_) were 0.85, 0.96 and 0.86, respectively.

The ROC curve analysis of the anthropometric parameters for the 1-year survival and their optimal cut-offs are summarized in Table 2. All 3D anthropometric parameters were statistically significant: VFM_3D_ (AUC = 0.554, *p* = 0.02, cutoff = 0.72 kg/m^2^), SFM_3D_ (AUC = 0.544, *p* = 0.047, cutoff = 3.05 kg/m^2^), FBM_3D_ (AUC = 0.550, *p* = 0.03, cutoff = 4.32 kg/m^2^) and MBM_3D_ (AUC = 0.565, *p* = 0.007, cutoff = 5.47 kg/m^2^). Whilst only the VFA_2D_ (AUC = 0.548, *p* = 0.034, cutoff = 22.20 cm^2^/m^2^) was statistically significant for the 2D parameters.

**Figure 3:**
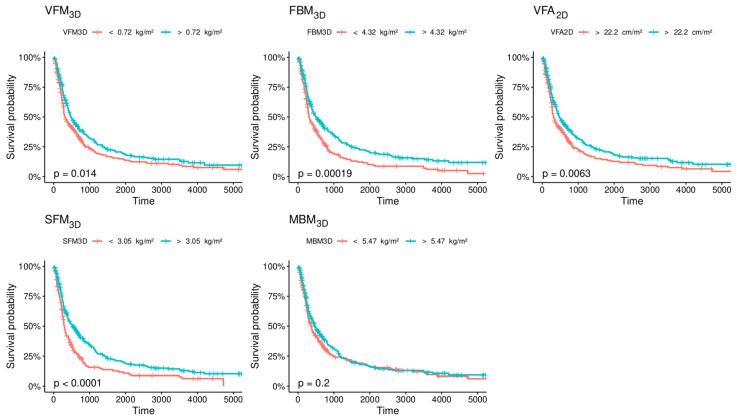
Kaplan–Meier estimates of overall survival (OS) according to the optimal cut-offs of VFM_3D_ (cutoff = 0.72 kg/m^2^), SFM_3D_ (cutoff = 3.05 kg/m^2^), FBM_3D_ (cutoff = 4.32 kg/m^2^) MBM_3D_ (cutoff = 5.47 kg/m^2^) and VFA_2D_ (cutoff = 22.2 kg/m^2^).

The Kaplan–Meier estimates of survival according to VFM_3D_, SFM_3D_, FBM_3D_, MBM_3D_ and VFA_2D_ are shown in Figure 3. Using the optimal cut-offs, most 3D parameters significantly separated the OS of the populations (log-rank test *p*-value: 0.014, <0.0001, 0.0002, 0.0063 for VFM_3D_, SFM_3D_, FBM_3D_ and VFA_2D,_ respectively). MBM_3D_ was not significantly associated with the OS (*p* = 0.2).

### 3.3. Sub Analyses

**Figure 4:**
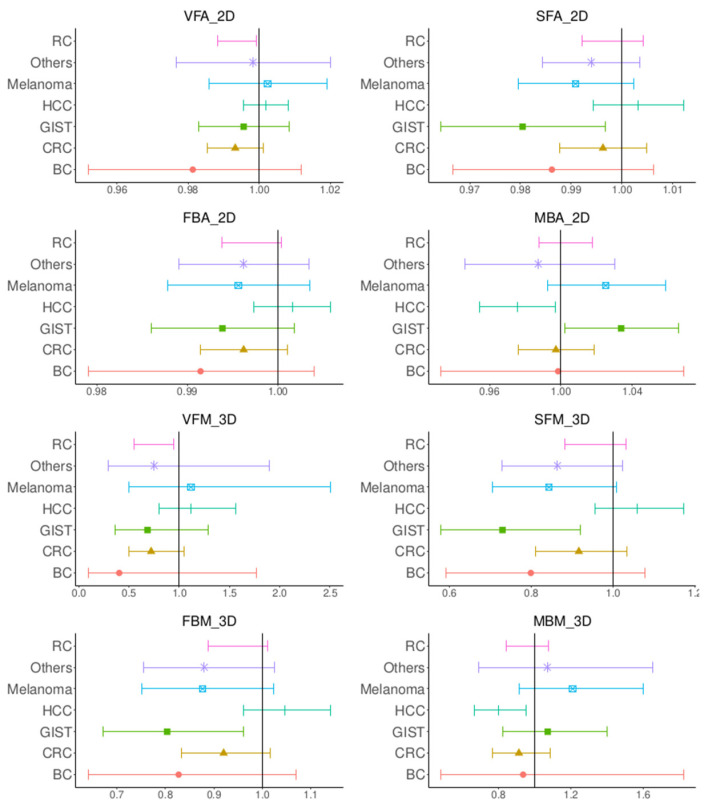
Forest plots of the log hazard ratios computed using Cox regression models by stratifying according to cancer types for VFA_2D_, SFA_2D_, FBA_2D_, MBA_2D_, VFM_3D_, SFM_3D_, FBM_3D_, MBM_3D_.

The analysis of the anthropometric parameters after stratification by gender using univariate Cox regression models is shown in Table 3. For women, none of the 3D or 2D anthropometric parameters were statistically significant (Null hypothesis: Hazard ratio = 1, *p* > 0.05). However, for men, all the anthropometric parameters except MBA_2D_ were statistically significant. Cox regression models were also used to evaluate the anthropometric parameters in a univariate study of the population stratified by cancer type Figure 4. VFM_2D_ and VFM_3D_ were statistically significant for renal cancers, SFM_3D_, SFA_2D_ and FBM_3D_ for GIST and MBA_2D_ and MBM_3D_ for HCC (with a paradoxical effect of MBA_2D_ for GIST).

## 4. Discussion

In this study, we investigated the contribution of body composition in determining the prognosis in a large population (526 patients) of multiple cancer types treated with targeted therapy. We compared three-dimensional and two-dimensional automatic measurements and found a better overall value for the three-dimensional parameters, especially on the ROC curves where all the 3D parameters (SFM_3D_, VFM_3D_, FBM_3D_ and MBM_3D_) were statistically significant in the whole population. For the subgroup analyses, a significant prognostic value was found in males. Whereas for cancer type, VFM_3D_ was statistically significant for renal cancers, SFM_3D_ and FBM_3D_ for GIST and MBM_3D_ for HCC.

The use of three-dimensional rather than two-dimensional segmentation to determine body composition is novel [16,25] and provides more accurate measurements than the two-dimensional segmentation computed at the L3 abdominal level [16,26]. This difference may explain why the 3D parameters were significant while the 2D parameters were not. Moreover, a paradoxical result was obtained: For GIST cancers, the 2D parameter of the muscle area (MBA_2D_) suggests that a large muscle area is related to a worse prognosis, while the 3D parameter (MBM_3D_) suggests the opposite. Compared to the 2D measurements and other 3D software [25], one of the strengths of the 3D analysis performed by Anthropometer3DNet is that it uses factors for extrapolation of the data outside the field of acquisition. It can obtain a total mass rather than an area or an index in the results, and this global mass measurement could potentially be useful for therapeutic adaptation. Moreover, thanks to the automatic segmentation performed and the wide windowing of the Hounsfield units, it is exploitable on injected and non-injected scanners. For research, the Anthropometer3DNet software is available on the Oncometer3D.com platform via an online service.

In this study, there were some differences in the results between the different populations in the sub-analyses. For example, VFM_2D_ and VFM_3D_ were statistically significant for renal cancers, SFM_3D_, SFA_2D_ and FBM_3D_ for GIST and MBA_2D_ and MBM_3D_ for HCC, while the other cancer types had no significant parameter. This difference could be explained by the lack of statistical power due to the reduced number of patients in the subpopulations but also by the preponderant prognostic role of neoplasia for some types of cancer. Morever, the difference in body composition [27] might also be the reason some parameters were significant for men and not for women. 

In the subgroup analysis by cancer type, some results are similar to what has been shown in the literature. Firstly, visceral fat was a prognostic factor for kidney cancer [28]. Secondly, S. Antoun and al. showed that low BMI (<25 kg/m^2^) with diminished muscle area was a significant predictor of toxicity in metastatic RC patients treated with sorafenib [11], muscle loss being specifically exacerbated by this treatment [29]. Moreover, we found that muscle was a prognostic factor for HCC which has already been documented [30]. The subcutaneous and total fat (which are highly correlated) are prognostic factor for GIST which has not yet, to our knowledge, been documented in the literature, possibly due to the relative rarity of this disease.

This study has some limitations. Its retrospective nature may have led to a lack of data. Thus, the weight of the patients, a variable parameter according to time, was not systematically available in a period corresponding to the realization of the CT examination which did not allow us to compare the body composition parameters with BMI. Furthermore, although the population is large (corresponding to cancers treated with one type of targeted therapy), it is nevertheless heterogeneous, which explains the relatively moderate, albeit statistically significant, overall prognostic values and the differences observed in the subgroup analyses, by gender and by type of cancer. Thus, rather than being used in the general population, body composition may be best used for specific types of cancers and patients. 

Finally, the use of body composition to determine prognosis, using fat compartments, as shown in this study, could be useful to choose therapeutic modalities or adapt dosages to the morphotype. The physiopathological principles remain to be determined further in pre-clinical and/or prospective studies. Moreover, if body composition corresponds to a description of the tumor host, its association with parameters describing the tumor could be of interest to better specify the prognosis. This could, for example, be associated with a radiomic analysis using artificial intelligence models, as performed by Schutte et al. on the same database, combining ultrasound images, CT images and clinical data [31].

## 5. Conclusions

Automatic and three-dimensional body composition on pre-therapeutic CT scans is feasible and can improve prognostication in patients treated with antiangiogenic drugs. Three-dimensional measurements are more effective than two-dimensional measurements.

## Figures and Tables

**Table 1:** Characteristics of the patients.

Characteristic	Patients (N = 526)
Sex, *n* (%)	
Male	377 (71.7%)
Female	149 (28.3%)
Age *	
Median	58
Range	[19–83]
Tumor, *n* (%)	Tumor, *n* (%)
Renal cell carcinoma	204 (38.8%)
Colorectal carcinoma	93 (17.7%)
Hepatocellular carcinoma	72 (13.7%)
Gastrointestinal stromal tumor	56 (10.6%)
Melanoma	42 (8.9%)
Breast cancer	27 (5.1%)
Others	32 (6.1%)
Antiangiogenic treatment, *n* (%)	
Bevacizumab	137
Sunitinib	117
Sorafenib	107
Axitinib	34
Imatinib	32
Other	101

* Data were missing for 2 patients.

**Table 2:** Diagnostic performance of clinical and anthropometric parameters measured on the CT for OS using a ROC analysis for the prediction of survival at 1 year. Optimal cut-offs were determined using Youden’s index.

	Mean *Median* (+/−SD) [Min-Max]	Cut-Off Value	AUC	Sensitivity	Specificity	Accuracy	*p*-Value
Line of treatment	2.07 *2* (±1.47) [1–10]	2	0.63	0.61	0.60	0.60	< 0.001
VFM_3D_	0.85 kg/m^2^ *0.75 kg/m^2^* (+/−0.62) [0.04–3.25]	0.72	0.554	0.56	0.54	0.55	0.02
SFM_3D_	4.18 kg/m^2^ *3.88 kg/m^2^* (+/−2.27) [0.13–13.87]	3.05	0.544	0.73	0.41	0.57	0.047
FBM_3D_	5.03 kg/m^2^ *4.70 kg/m^2^* (+/−2.70) [0.17–16.12]	4.32	0.550	0.63	0.48	0.56	0.03
MBM_3D_	5.74 kg/m^2^ *5.65 kg/m^2^* (+/−1.35) [2.44–10.83]	5.47	0.565	0.60	0.49	0.58	0.007
VFA_2D_	36.85 cm^2^/m^2^ *30.01 cm^2^/m^2^* (+/−30.86) [0.01–161.91]	22.20	0.548	0.64	0.46	0.55	0.034
SFA_2D_	53.14 cm^2^/m^2^ *47.16 cm^2^/m^2^* (+/−30.86) [0.77–184.63]	NA	0.533	NA	NA	NA	0.10
FBA_2D_	89.99 cm^2^/m^2^ *86.07 cm^2^/m^2^* (+/−53.17) [2.52–243.48]	NA	0.543	NA	NA	NA	0.052

FBA_2D_: fat body area 2D; FBM_3D_: fat body mass 3D; MBA_2D_: muscle body area 2D; MBM_3D_: muscle body mass 3D; SFA_2D_: subcutaneous fat area 2D; SFM_3D_: subcutaneous fat mass 3D; VFA_2D_: visceral fat area 2D; VFM_3D_: visceral fat mass 3D.

**Table 3:** Univariate analysis using continuous values for global metrics and anthropometric parameters measured on CT-scan. The hazard ratio was computed using a Cox regression model.

	Whole Population (Men and Women)		Men		Women	
	HR	*p-*Value	HR	*p-*Value	HR	*p-*Value
Sex	0.96	0.70				
Age	1.00	0.395	1.00	0.97	1.00	0.58
Line of treatment	0.88	< 0.0001	1.28	< 0.0001	1.14	0.0067
VFM_3D_	0.55	0.12	0.73	0.0026	0.87	0.52
SFM_3D_	0.98	0.46	0.93	0.036	0.94	0.11
FBM_3D_	0.99	0.31	0.93	0.014	0.95	0.14
MBM_3D_	0.93	0.05	0.89	0.024	0.85	0.14
VFA_2D_	1.00	0.33	0.99	0.015	1.00	0.48
SFA_2D_	1.00	0.47	0.99	0.027	1.00	0.47
FBA_2D_	1.00	0.33	0.997	0.009	1.00	0.44

## Data Availability

Not applicable.
